# Advancing flexible optoelectronics with III-nitride semiconductors: from materials to applications

**DOI:** 10.1038/s41377-025-02052-0

**Published:** 2026-03-03

**Authors:** Xingfa Gao, Yuzhen Huang, Rixuan Wang, Yinglun Sun, Lai Wang

**Affiliations:** 1 Institute of Medical Engineering and Interdisciplinary Research, Medical Science and Technology Innovation Center, Shandong First Medical University & Shandong Academy of Medical Sciences, 250117 Jinan, China; 2 Medical Engineering and Technology Research Center, School of Radiology, Shandong First Medical University & Shandong Academy of Medical Sciences, 271016 Taian, China; 3 State Key Laboratory of Widegap Semiconductor Optoelectronic Materials and Technologies, and Department of Electronic Engineering, Tsinghua University, 100084 Beijing, China

**Keywords:** Optoelectronic devices and components, Optical materials and structures

## Abstract

The rapid evolution of wearable technology, interconnected devices, and medical devices is driving innovation in advanced materials for flexible optoelectronics. III-nitride semiconductors, with their exceptional optoelectronic properties, strong piezotronic and piezo-phototronic effects, biocompatibility, and thermal/chemical/mechanical stability, present a compelling alternative to traditional organic and Si-based inorganic materials. Despite significant research efforts, a systematic review summarizing the advancements and challenges in III-nitride flexible optoelectronics is lacking. This article provides a comprehensive overview of recent developments in this field. It begins by highlighting the advantages of III-nitride semiconductors for flexible optoelectronics. The article then discusses the fabrication techniques for III-nitride flexible devices, covering materials growth, film exfoliation and transfer, as well as functional micro/nanostructures. A wide range of flexible applications of III-nitrides are explored, including flexible displays, implantable optogenetic devices, wearable photodetectors, and flexible mechanical sensors. Finally, challenges and potential solutions related to device fabrication, performance enhancement, theoretical modeling, and system integration are discussed. This work serves as a foundational reference and roadmap for further advancements in III-nitride flexible optoelectronics.

## Abstract (web summary)

Rooted in superior material properties, III-nitride flexible optoelectronics thrive across flexible applications via advanced material growth and transfer processes.

## Graphical abstract

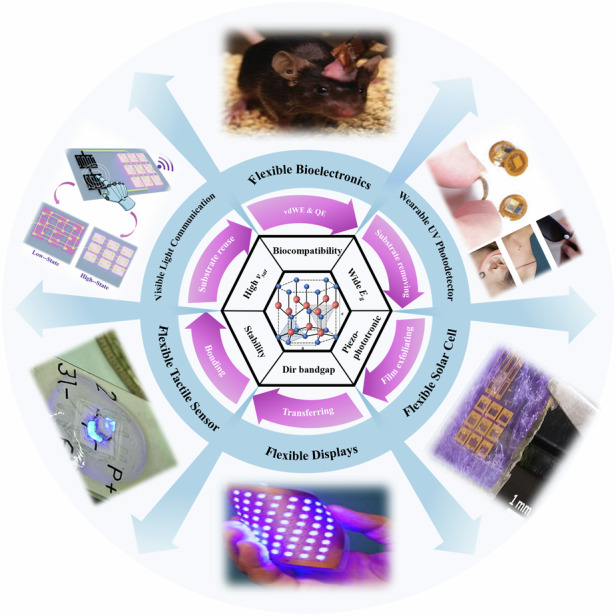

## Introduction

The evolutionary demand for dynamic adaptability of optoelectronic devices across consumer electronics, human-machine interfaces, visual Internet of Things (IoT), wearable/implantable medical systems, and defense-specific applications drives the transition toward flexible optoelectronics^1,2^. By integrating conventional optoelectronic functionalities with mechanical flexibility, this field unlocks substantial market scalability. Beyond innovations in device architecture and manufacturing processes, systematic material-level innovations will accelerate the iterative evolution of flexible optoelectronics^3^. Although organic materials have achieved notable progress in flexible optoelectronics owing to intrinsic flexibility, low cost, and large-area processability^4^, they often suffer from inherent limitations such as slow response times, constrained environmental stability, and inadequate long-term durability^5,6^. Inorganic semiconductors present viable solutions to these challenges, particularly as advances in flexible device manufacturing over recent decades have established a robust foundation for inorganic flexible optoelectronics^7^. Among these, III-nitrides, notably gallium nitride (GaN), aluminum nitride (AlN), indium nitride (InN), and their ternary alloys, have emerged as leading candidates in the third-generation semiconductor landscape, outperforming previous generations of semiconductors in multiple critical aspects. These materials possess distinctive properties: widely tunable direct band gaps, high electron saturation velocities (*v*_*sat*_), low dielectric constants (*ε*), strong spontaneous and piezoelectric polarization fields, exceptional robustness, environmental stability, and biocompatibility^8^. Furthermore, their mature fabrication protocols enable high-performance optoelectronic devices (e.g., LEDs, photodetectors, solar cells, and lasers) with exceptional quantum efficiencies^9^. The singular material and device characteristics of III-nitrides unlock transformative pathways for advancing flexible optoelectronics^10^.

**Fig. 1:**
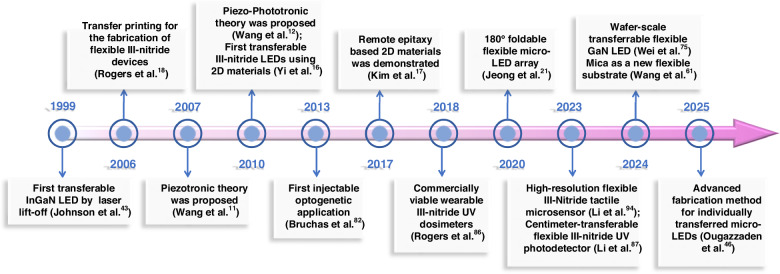
Historical trajectory of key milestones in III-nitride flexible optoelectronics

**Fig. 2:**
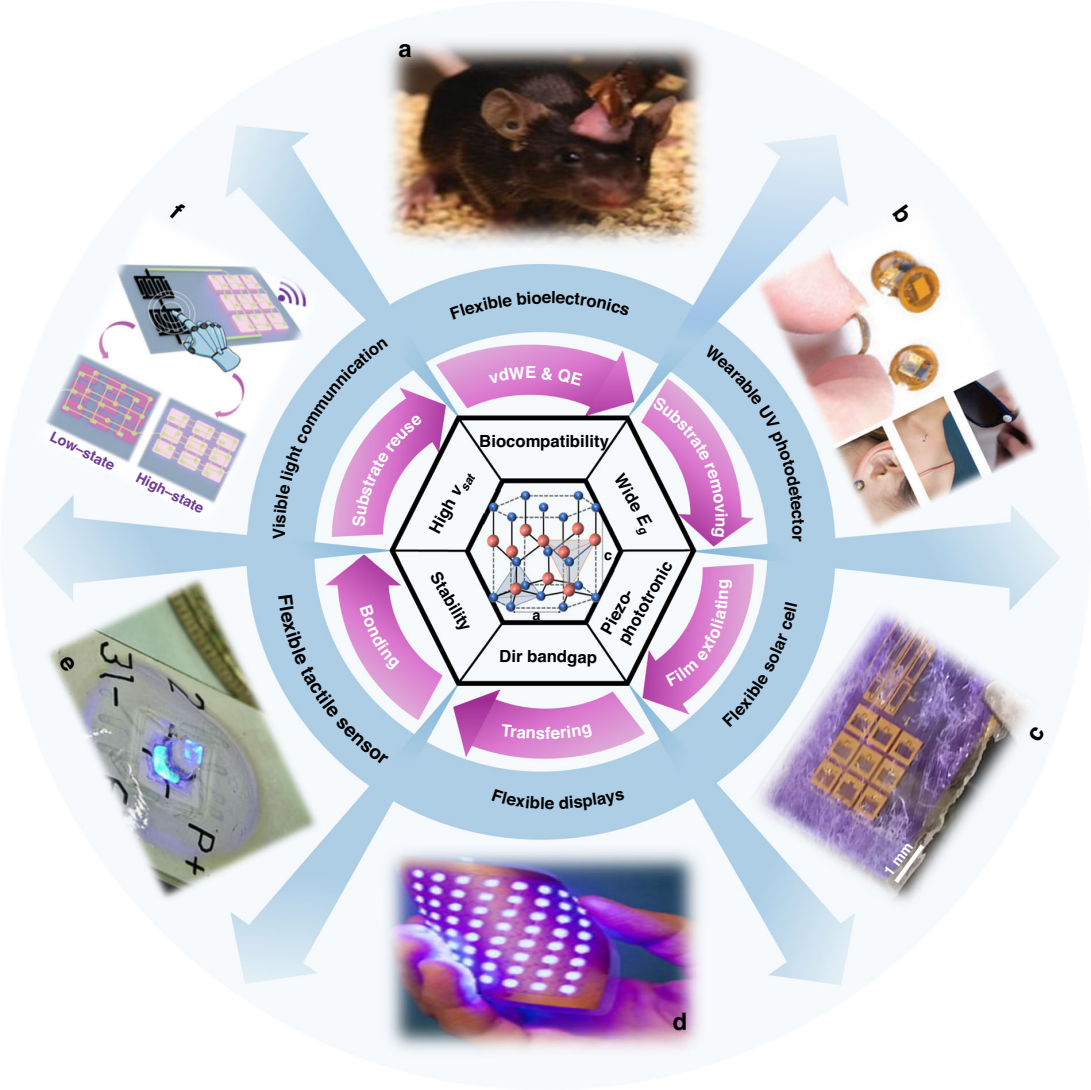
Schematic outline of this review. The material properties are used as a starting point for the fabrication technologies and the development of the featured flexible applications. **a** A custom flexible optoelectronic device mounted on freely moving animals for wireless optogenetic application. Reproduced with permission from ref. ^82^. Copyright 2013, American Association for the Advancement of Science. **b** Wearable wireless sensors for millimeter-scale, battery-free UVA radiation application in body parts. Reproduced with permission from ref. ^86^. Copyright 2018, The American Association for the Advancement of Science. **c** Photograph of InGaN-based solar on glass substrates with piezo-phototronic effect enhanced performance. Reproduced with permission from ref. ^96^. Copyright 2018, American Chemical Society. **d** Mechanical properties of the LEDs for flexible display. Reproduced with permission from ref. ^77^. Copyright 2013, Elsevier. **e** GaN optical three-axis tactile microsensor mounted on a flexible cap layer. Reproduced with permission from ref. ^94^. Copyright 2023, IEEE **f** Schematic diagram of the self-variable-voltage optical communication systems. Reproduced with permission from ref. ^97^. Copyright 2014, Elsevier

Figure 1 summarizes key developmental milestones in III-nitride flexible optoelectronics. Since the introduction of piezotronics and piezo-phototronics by Wang, the use of mechanical deformation to enhance the performance of piezoelectric semiconductors has unlocked the enormous potential of III-nitride flexible optoelectronics^11–13^. Advances in fabrication technology have further accelerated the integration of III-nitride semiconductors in flexible optoelectronics^14,15^. For instance, Yi et al.^16^ demonstrated GaN thin film growth on graphene via van der Waals epitaxy (vdWE), while Kim et al.^17^ validated the viability of remote epitaxy (RE), expanding the horizons of material growth on unconventional substrates. Transfer printing has facilitated efficient device transfer processes^18^. Further advancements in substrate removal, film exfoliation, and transfer techniques have provided critical pathways for fabricating flexible III-nitride devices^19^. The incorporation of micro/nanostructures has greatly improved the mechanical flexibility and functionality of these devices^20,21^. As illustrated in Fig. 2, advances in technology have paved the way for the integration of III-nitride semiconductors into flexible optoelectronics, thus sparking the generation of a range of innovative devices. Recent years have witnessed significant advancements in diverse emerging applications, particularly in flexible displays, implantable optogenetic devices, wearable photodetectors, and flexible mechanical sensors.

Despite significant progress in III-nitride flexible optoelectronics, it still faces considerable challenges in the flexible fabrication techniques as well as practical applications. This review offers the first comprehensive analysis of the advantages, advancements, challenges, and future prospects of III-nitride flexible optoelectronics. We begin by exploring the properties of III-nitride semiconductor materials and their superior performance in flexible optoelectronics. Next, we summarize the evolution of flexible fabrication techniques, including vdWE, RE, film exfoliation and transfer techniques, and the development of micro/nano-functional structures. The review then highlights diverse applications of III-nitride flexible optoelectronics. Finally, we discuss the key challenges and propose potential solutions, offering insights into future research directions. This review aims to provide a valuable resource to advance the development of III-nitride flexible optoelectronics.

## Advantages of III-nitride semiconductors for flexible optoelectronics

**Fig. 3:**
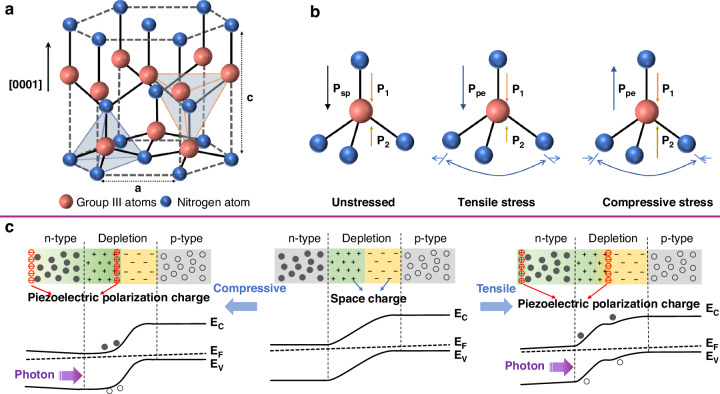
Piezoelectric properties of III-nitrides. **a** Schematic of III-nitride wurtzite crystal structure. **b** Schematic illustration of the piezoelectric polarization (*P*_*pe*_) effect in metal-polar III-nitrides^30^. **c** Schematic of energy band profile with the effect of piezo-phototronic effect on the photo-response of a PN junction under compressive and tensile strains^13,14^

Organic semiconductors leverage intrinsic flexibility and solution-processability to enable cost-effective, ultrathin, and lightweight flexible optoelectronic devices. Consequently, they have gained significant traction in applications such as low-cost solar cells and foldable displays^22^. Despite commercial successes in applications such as small-scale mobile displays, broader implementation is constrained by inherent limitations: low charge carrier mobility (typically ~10 cm^2^/V·s)^23^, environmental instability (performance degradation under air/moisture/light exposure)^10^, and short operational lifetimes (e.g., ~4 years for organic light emitting diodes (OLEDs))^6^. III-nitride semiconductors have emerged as pivotal materials in inorganic optoelectronics^8^. They exhibit exceptional carrier mobility, with two-dimensional electron gas (2DEG) mobility at heterojunctions exceeding 2000 cm^2^/V·s^24^. Their wide bandgap, stable crystal structure, and robust chemical bonds confer outstanding mechanical, thermal, and chemical stability under diverse environmental conditions. Consequently, devices based on III-nitrides achieve extended operational lifetimes (InGaN/GaN LEDs typically exceed 10 years)^6^. Such capabilities directly address the deficiencies of organic semiconductors, thereby expanding the application frontiers of flexible optoelectronics^10^. In addition, a comparison of the key physical parameters of inorganic semiconductor materials presented in Table 1, illustrates the distinct advantages of III-nitrides. III-nitrides (except for InN) offer the widest bandgap, the highest *v*_*sat*_, and the strongest breakdown field strength. These materials also boast a direct bandgap, high thermal conductivity, low *ε*, strong spontaneous and piezoelectric polarization effects^8^. As shown in the wurtzite crystal structure in Fig. 3a, considerable ionic bonding components are due to the large electronegativity difference between the elements. The bond length along the [0001] direction is longer than that of the other three bonds, displacing the positive and negative electrostatic centers and creating a strong spontaneous polarization along the polar axis^25^. Figure 3b illustrates the generation of piezoelectric polarization in metal-polar III-nitrides. The remarkable properties of III-nitrides have driven the development of a wide array of optoelectronic devices, including LED^26^, photodetector^27^, solar cell^28^, and laser diode^29^. III-nitride semiconductors operate in the entire spectrum, from deep UV to IR. Additionally, both n-type and p-type conductivity can be achieved in III-nitrides. Since the advent of GaN-based blue LEDs and their subsequent commercial success, multi-band nitride LEDs have evolved into a vital component of solid-state lighting due to their high efficiency, long lifespan, and environmental friendliness.

**Table 1:** Key physical parameters of Si, GaAs, 4H-SiC, GaN, and AlN materials

Physical parameters/units	Si	GaAs	4H-SiC	GaN	AlN
Bandwidth *E*_*g*_ */eV*	1.1	1.4	3.3	3.5	6.2
Electron saturation velocity *v*_*sat*_ */(10*^*7* ^*cm/s)*	1.0	2.0	2.0	2.7	1.4
Breakdown field strength *E*_*C*_ */(MV/cm)*	0.3	0.4	3.0	3.3	15.0
Direct bandgap	-	√	√	√	√
Thermal conductivity *κ /[W/(cm·K)]*	1.5	0.5	4.9	2.3	3.4
Relative dielectric constant *ε*	11.4	13.1	9.7	8.9	8.5

Traditional III-nitride devices, fabricated on rigid substrates like Si, SiC, and sapphire, have seen remarkable advancements and are now widely commercialized. In recent years, III-nitride flexible optoelectronics have been particularly attractive as emerging applications move toward flexibility, endurance of mechanical deformation, multi-operating environment adaptability, and implantability. Notably, the theoretical foundations established by Wang’s group on piezotronic and piezo-phototronic effects have paved the way for the novel phenomena exhibited by III-nitride semiconductors in flexible devices^14^. The piezo-phototronic effect merges the features of semiconductors, piezoelectric polarization, and optical excitation. The piezoelectric potential resulting from mechanical strain can influence carrier generation, transport, separation, and recombination, thus improving the functionality of flexible optoelectronic devices. Figure 3c illustrates the impact of the piezo-phototronic effect on the photo-response of a PN junction. When strain is introduced, the residual piezoelectric polarization on the n-type semiconductor side influences both the width and the shape of the depletion region, as well as the local band alignment at the junction^30^. This modulation plays a crucial role in redistributing photogenerated electrons and holes. Similar effects can be extended to more intricate scenarios^13,31^.

Based on the above-mentioned development of materials and devices, III-nitrides are gradually revealing many advantages in flexible optoelectronics^13,19,32,33^. (ⅰ) III-nitride optoelectronic devices, with their high light emission and photoelectric conversion efficiency, long lifespans, and environmental friendliness, offer promising options for applications in flexible lighting, displays, and detection. The direct tunability of their bandgap allows them to operate across the entire spectrum, thereby enhancing the multifunctionality and integration of flexible applications based on III-nitride systems. (ⅱ) The inherent stability and biocompatibility of III-nitride materials enable them to form direct, functional interfaces with biological tissues. This biocompatibility, combined with their mechanical robustness, thermal, and chemical stability, underscores the significant potential of III-nitride materials in challenging environments, as well as in wearable and implantable technologies. (ⅲ) The pronounced piezo-phototronic effects not only enhance the photoresponsivity of flexible optoelectronic devices but also enable sensitive mechanical responses and self-powered operation. (ⅳ) The high *v*_*sat*_ and low *ε* enable dynamic responses and fast response times in flexible devices.

## Material growth and fabrication techniques

The unique properties of III-nitrides, combined with versatile device architectures, present exciting opportunities for flexible optoelectronics. However, a key challenge in this area lies in their mechanical stiffness, stemming from their intrinsically high Young’s modulus and the use of thick, rigid substrates. To address this, flexible substrate-based material growth and fabrication processes have received extensive attention. The development of vdWE, RE, advanced film lift-off and transfer techniques, as well as micro/nanostructure engineering, has offered viable technological pathways for implementing flexible optoelectronics, thereby unlocking the inherent advantages of III-nitride semiconductors in flexible devices.

### Van der Waals epitaxy and remote epitaxy

The fabrication of flexible semiconductor devices primarily employs two strategies: direct growth on flexible substrates and post-growth transfer from rigid substrates. However, direct epitaxial growth of III-nitrides faces significant challenges due to the thermal instability of flexible amorphous substrates at high temperatures (>1000 °C) required for high-quality growth, coupled with the critical need for precise lattice and thermal matching^20,34^. Alternatively, the post-growth transfer method addresses these limitations but requires buffer layer integration to overcome the strong sp^3^-type covalent bonds between conventional rigid substrates and epilayers, enabling effective layer detachment. 2D crystalline materials, consisting of single-atom or single-molecule thick layers, provide a promising platform for flexible devices. These materials feature strong intralayer covalent bonding and weak interlayer vdW forces, exhibiting several advantageous characteristics: (ⅰ) high thermal stability compatible with III-nitrides growth conditions, (ⅱ) dangling bond-free surfaces enabling effective vdWE and RE, (ⅲ) weak interlayer interactions facilitating layer separation, and (ⅳ) unique intrinsic properties, including tailored electrical, thermal, optical, magnetic, and mechanical characteristics surpassing conventional bulk substrates^35^.

**Fig. 4:**
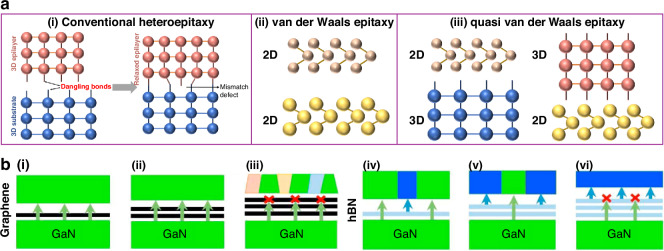
VdWE and QE. **a** Schematic representation of the conventional heteroepitaxy, vdWE and quasi vdWE^37^. **b** Schematic of the growth mode of the RE GaN on graphene/h-BN. Reproduced with permission from ref. ^40^. Copyright 2018, The Author(s), under license to Springer Nature

VdWE denotes a heterogeneous growth that capitalizes on the weak vdW forces rather than robust chemical bonding^36^. As shown in Fig. 4a, conventional heterogeneous epitaxy is prone to introducing strain and lattice defects. In vdWE, the weakly bound heterogeneous interface cannot significantly alter the first few layers of the epilayer and prevents the nucleation of defects even in the presence of large lattice mismatches^37^. In addition to growing on layered materials, bulk substrates can also be employed, provided their top surface is properly passivated^38^. The 3D/2D and 2D/3D cases were also known as quasi-vdWE^39^. Some studies focus solely on using 2D materials as seed layers for vdWE, detached from the substrate lattice. In addition, Kim et al. proposed that while 2D materials like graphene can serve as seed layers for vdWE, interactions with the substrate beneath the 2D materials still influence epilayer growth. Graphene, due to its osmotic transparency and weak vdW forces, does not fully shield the lattice potential field from the substrate, defining the mechanism as RE (Fig. 4b)^17,40^. With numerous studies on vdWE and RE^34^, high crystal quality III-nitride semiconductors are now available on 2D materials. This approach addresses substrate lattice and thermal mismatches to a certain extent while offering flexibility in substrate selection for preparing III-nitride flexible devices.

### Film exfoliation and transfer techniques

**Fig. 5:**
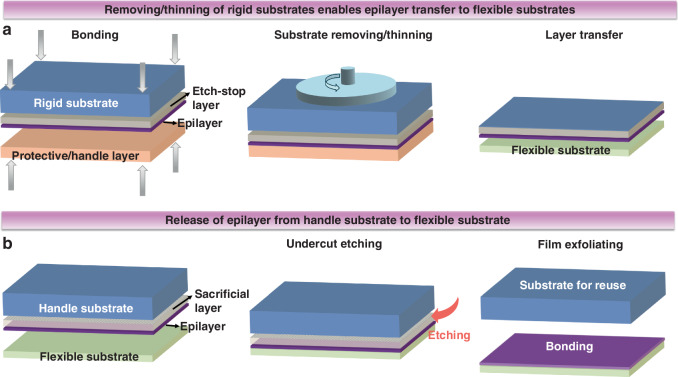
Schematic of the process. **a** To remove/thin the rigid substrate and **b** to exfoliate the epilayer from the rigid substrate

Flexible optoelectronics using inorganic semiconductor materials typically employ two primary fabrication approaches: (i) Device fabrication before exfoliating epilayers from rigid substrates (referred to as “Device-first”) and (ii) Device fabrication after transferring/depositing thin films onto flexible substrates (“Device-last”). Due to the high deposition temperatures and the challenges associated with subsequent high-temperature processing, the “Device-first” strategy has become the prevailing approach in flexible III-nitride optoelectronics. This approach enables flexible chips to retain the advantages of rigid counterparts, including fine feature sizes, dense integration, multilevel interconnects, complex circuit functionalities, and reliable operation. The main fabrication techniques and their corresponding advantages and disadvantages are shown in Table 2. The key step following device fabrication on origin substrates is to impart flexibility to the bulk material, which necessitates removing/thinning the rigid substrate or exfoliating the films from the rigid substrate. Removal of the rigid substrate can be accomplished by backside grinding, polishing, and etching to reduce the thickness of the substrate from the backside surface (Fig. 5a). However, the method of exfoliating the detached film from a large wafer retains the seed substrate for reuse^41^. Methods include chemical undercut etching, laser lift-off (LLO), transfer printing, and transferring based on 2D buffer layers.

**Table 2:** Main fabrication techniques and their corresponding advantages and disadvantages for flexible optoelectronics based on III-nitride semiconductors

Methods		Advantages	Disadvantages
*Backside grinding & polishing*		High removal rate, simple process, low cost, and large size compatibility	Easily causes surface and wafer damage, and imprecise thickness control
*Etching substrate*	*Dry etching*	Anisotropic etching, low stress to avoid damage and reduce warping, high precision thickness control	High process difficulty (especially for high-hardness substrates such as GaN and SiC), poor surface morphology
	*Wet etching*	Low cost, no mechanical stress, good surface uniformity	The etch rate difficult to control precisely, isotropic etching requires strict protection of front-side devices
*Selective lateral chemical etching*		Sacrificial layers enable easy substrate peeling without wafer damage, can be used for large-area peeling, low cost.	Requires advance preparation of specific sacrificial layers, increases epitaxial layer growth issues
*Laser lift-off*		High processing accuracy, low damage from ultra-fast lasers, high material utilization, no need for special protective layers	High costs and low efficiency of ultra-fast lasers, difficulty in processing large areas and controlling yield rates
*Transfer printing*		High-precision heterogeneous integration, damage-free transfer	Complex printing ink preparation process, high technical barriers, low point-by-point transfer efficiency
*Transferring based on 2D buffer layers*	*Self-separation*	Simple operation process, no additional materials required, convenient to transfer technology	2D layers prone to delamination, and defects or cracks easily introduced into the epilayer
	*Thermal release tap*	Easy to operate, highly efficient, low cost of tape, and reusable substrate	Uncontrollable, epilayer prone to damage, additional process required to remove tape
	*Mechanical spalling*	Precise and controlled exfoliation, metal stress sources provide support and protection for the epitaxial layer	Specific stress source layers need to be bonded and removed, complicated process, and costly

**Fig. 6:**
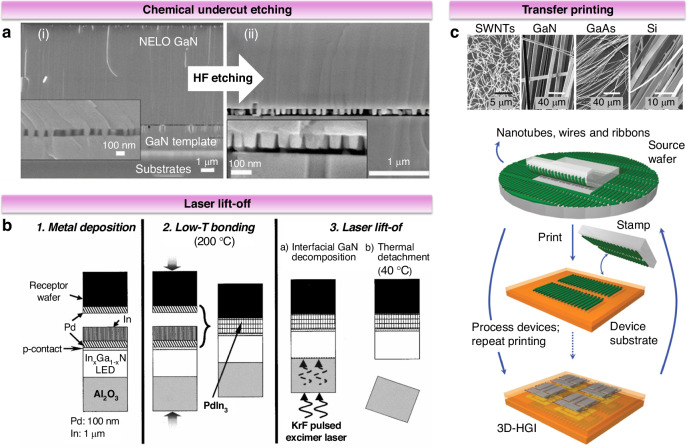
Rigid substrate removal and film transfer techniques. **a** SEM images of the nano-epitaxial lateral overgrowth (NELO) GaN epilayers before chemical release (ⅰ) and after chemical release (ⅱ). Reproduced with permission from ref. ^42^. Copyright 2010, The Authors, published by Springer Nature. **b** Process flow for transfer of InGaN LED by LLO. Reproduced with permission from ref. ^43^. Copyright 2000, AIP Publishing. **c** Schematic illustration of a 3D heterogeneous integration method based on transfer printed semiconductor nanomaterial. Reproduced with permission from ref. ^18^. Copyright 2006, American Association for the Advancement of Science

Exfoliation of III-nitrides can be accomplished through selective lateral chemical etching of a sacrificial layer placed between the rigid substrate and the layer intended for transfer (Fig. 5b). Zang et al. developed a simple wet chemical undercut etching method to release III-nitride films^42^. The method was based on a nano-epitaxial lateral overgrowth process (Fig. 6a). The scanning electron microscope (SEM) results showed that the GaN epilayers provided have low dislocation density, which is favorable for flexible optoelectronic devices. LLO is a technology that uses a laser for rapid exfoliation between a substrate and an epilayer, which is characterized by low damage and high precision. This method is particularly effective for hard materials like sapphire, which lack an efficient wet etchant. By carefully controlling laser energy and irradiation time, thermal stresses are induced at the substrate-epilayer interface, causing the epilayer to peel off. Wong et al. successfully demonstrated KrF pulsed excimer LLO of InGaN LEDs from sapphire substrates (Fig. 6b)^43^, while maintaining unchanged electrical and spectral characteristics. To further support the fragile epitaxial film and prevent the performance degradation after LLO, the introduction of a leveling layer is effective^44^.

In parallel, Ahn et al. has made significant strides in developing transfer printing techniques, enabling the heterogeneous integration of electronic systems in 2D or 3D configurations^18^. This method synthesizes different semiconductor nanomaterials on various substrates and uses transfer printing to combine them onto flexible platforms. As shown in Fig. 6c, by employing reusable soft stamps, semiconductor nanomaterials can be efficiently transferred, enabling the fabrication of heterogeneous electronics. The core of this process is a highly parallel protocol for printing ‘ink’. III-nitride semiconductor inks in various complex forms are produced from wafers using methods akin to those for fabricating Si micro/nanostructures^45^. This versatile technique enables spatial organization and functionality of micro and nanomaterials in 2D or 3D layouts, making it an invaluable tool for flexible optoelectronics. The ability to integrate diverse materials on curvilinear and flexible substrates holds immense potential for applications in flexible technologies^46,47^.

**Fig. 7:**
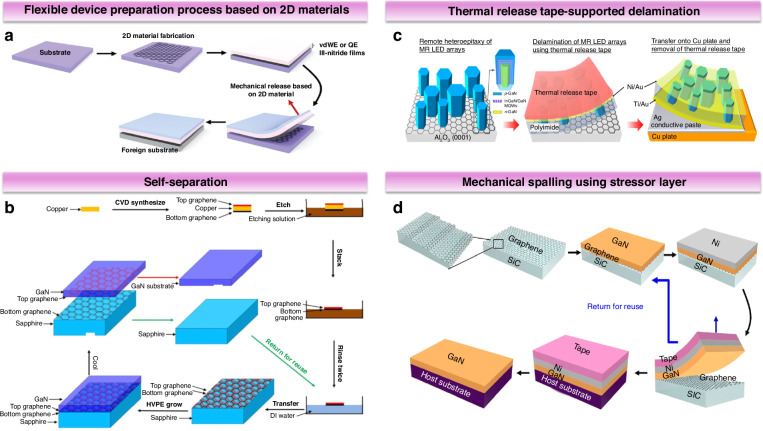
Film transfer techniques based on 2D materials. **a** Schematic for preparing III-nitride flexible films based on 2D materials. Reproduced with permission from ref. ^34^. Copyright 2019, Wiley-VCH GmbH. **b** Process of fabricating a self-separation GaN free-standing substrate. Reproduced with permission from ref. ^48^. Copyright 2020, Elsevier. **c** Process of delamination of GaN microarray LEDs using thermal release tape. Reproduced with permission from ref. ^21^. Copyright 2020, The Authors, published by American Association for the Advancement of Science. **d** Process for growing and transferring single-crystalline GaN thin films by a Ni stressor layer. Reproduced with permission from ref. ^49^. Copyright 2014, Springer Nature

Section 3.1 introduces vdWE and RE, and this part focuses on the 2D material-based exfoliating process (Fig. 7a). The weak connection between the epilayer and the 2D material makes it easy to exfoliate and transfer from the substrate. Currently, three special techniques (Fig. 7b–d) are employed for exfoliating films using 2D materials: self-separation^48^, thermal release tape-supported delamination^21^, and mechanical spalling using a stressor layer^49^. In practical applications, the choice of method can be tailored based on the substrate type, the thickness and area of the epilayer, and the specific characteristics of the interface^19,34^.

### Functional micro/nanostructures

**Fig. 8:**
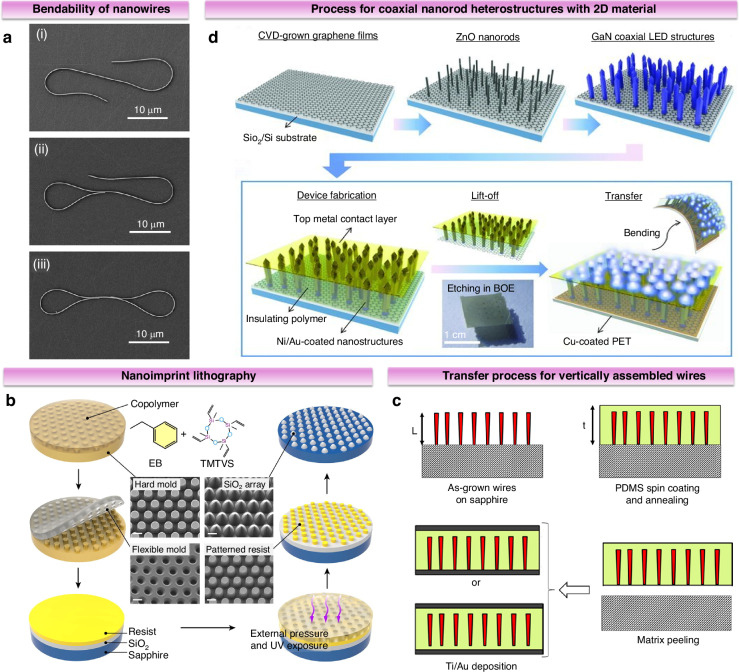
Micro/nanostructures as functional units to enhance mechanical flexibility. **a** SEM images of NWs in different bending states. Reproduced with permission from ref. ^50^. Copyright 2011, American Chemical Society. **b** Fabrication process of microstructure by flexible nanoimprint lithography and two-step etching process. Reproduced with permission from ref. ^15^. Copyright 2024 Science China Press. **c** Fabrication process flows for the realization of a flexible piezoelectric sensor with vertically assembled wires. Reproduced with permission from ref. ^53^. Copyright 2018, American Chemical Society. **d** Schematic illustration of the process used to fabricate flexible LEDs using GaN/ZnO coaxial nanorod heterostructures grown on graphene films. Reproduced with permission from ref. ^55^. Copyright 2011 Wiley-VCH GmbH

Micro/nanostructures serve as functional units that can enhance the overall device’s mechanical flexibility. Such as the nanowires (NWs) in Fig. 8a exhibit multi-angle bendability^50^. With ongoing advances in semiconductor technology, device sizes have reduced substantially, boosting chip integration. As materials scale down to the micron or even nanoscale, their specific surface area increases, introducing effects that traditional solid-state or atomic physics may not fully capture, such as quantum effects, size effects, and surface phenomena. Micro/nanostructures outperform thin films in strain relaxation, tolerating larger lattice mismatches and bypassing epitaxial constraints to enable flexible device integration. For flexible optoelectronic devices, micro/nanostructures offer substantial advantages: (ⅰ) high crystal quality and surface area provide excellent light extraction and absorption efficiencies, and contribute to photon localization effects, (ⅱ) moderate polarization fields in active regions via strain relaxation, enhancing electron-hole wavefunction overlap and improving light-emission efficiency, (ⅲ) more sensitive to mechanical excitation compared to planar structures and can respond to multiple modes of stresses and strains.

Several methods have been developed to prepare III-nitride micro/nanostructures, generally classified into top-down micro/nanomachining and bottom-up growth strategies. Top-down methods rely on high-quality bulk materials, where final micro/nanostructures are formed by selectively etching target regions using techniques like photolithography or self-assembly on nanorod surfaces^51^. Recently, Cui et al. developed a direct nanoimprinting technique for biomimetic microstructure fabrication on flexible substrates (Fig. 8b)^15^. Utilizing a copolymer-based flexible mold with dual-phase etching protocol, the method produced compound-eye-inspired Al_2_O_3_/SiO_2_ templates, achieving 6.4-fold higher production yield with 25% cost reduction compared to conventional stepwise projection lithography. Bottom-up approaches start from individual molecules or atoms to build the desired micro/nanostructures, providing better flexibility in controlling the device structure. Key bottom-up growth techniques for III-nitrides include vapor-liquid-solid growth, catalyst-free spontaneous growth, and selective area epitaxy. Following structure preparation, transfer printing enables these micro/nanostructures to be placed onto flexible substrates. Since free-standing micro/nanostructures are typically anchored by only a few points, this transfer process is more straightforward than traditional thin-film transfer techniques^18^. Salomon et al. employed sonication in a solution of iso-octane and isopropanol to separate GaN NWs from the sapphire substrate, subsequently drop-coating them onto a flexible polyethylene naphthalate (PEN) film pre-patterned with electrodes for straightforward transfer^52^. Kacimi et al. used autocatalytic metal-organic vapor phase epitaxy to grow GaN NWs, which were embedded in polydimethylsiloxane (PDMS) and detached effortlessly, allowing for the fabrication of flexible capacitive structures upon metal deposition (Fig. 8c)^53^.

Notably, combining the advantages of 1D nanostructures with 2D materials represents a promising strategy for advancing III-nitride flexible optoelectronics. As previously discussed, these 2D materials enable vdWE/RE and substrate transfer, essential for fabricating flexible devices with reliable integration. The direct growth of 1D semiconductor nanostructures on 2D layers enables wafer-scale fabrication of flexible mixed-dimensional heterostructures in a single run, avoiding transfer processes and scaling limitations common with traditional 2D materials^54^. Lee et al. successfully synthesized GaN/ZnO coaxial nanorod heterostructures on graphene films, subsequently transferring them to plastic substrates (Fig. 8d)^55^. The integration of micro/nanostructures with metal films is another promising strategy, particularly for its potential to simplify device fabrication by eliminating the need for complex transfer methods and thereby increasing throughput. The high electrical conductivity and thermal dissipation offered by metal substrates can further enhance device performance. To develop scalable, low-cost, and high-power micro/nanostructured devices, significant progress has been made in the direct growth of high-density, high-quality micro/nanostructures on bulk metal substrates^56–58^.

### Technology outlook

#### Material growth

Despite successful growth via vdWE and RE, a standardized growth model is lacking. Understanding these mechanisms is vital for selecting 2D materials with optimized bonding strengths and diffusion barriers. Surface modification or developing 2D materials with enhanced surface energy could promote heterogeneous epitaxy. Combining conventional growth methods with vdWE and QE, along with optimized pre-growth processes, can significantly improve epitaxial quality^59^. Direct growth of 2D materials on bulk substrates requires reducing surface damage and improving interfacial integrity. Additionally, direct growth of III-nitride films on flexible substrates offers a more efficient route. More research can focus on high-temperature-resistant metals like tungsten, molybdenum, hafnium, and alloys, as well as promising substrates like quartz^60^ and mica^61^. Furthermore, optimizing low-temperature sputtering and MBE growth techniques is essential to overcoming current limitations^62,63^.

#### Fabrication techniques

Direct growth of materials on flexible substrates offers simplified fabrication by avoiding complex exfoliation and transfer processes. However, this approach remains primarily confined to laboratory-scale demonstrations due to two critical limitations: (1) Subsequent device fabrication steps, such as rapid thermal annealing, require high-temperature processing incompatible with most flexible substrates; (2) The propensity for wrinkling in flexible substrates introduces handling complications during device processing. In contrast, the “Device-first” strategy leverages well-established, rigid semiconductor manufacturing advantages, including fine feature patterning, standardized design protocols, and high fabrication yields, to enable reliable industrial-scale production. After device fabrication on rigid substrates, exfoliation and transfer to flexible platforms are implemented. Traditional grinding and dry/wet etching methods for substrate removal offer procedural simplicity; their destructive nature prevents reuse. This is especially problematic for expensive and high-hardness rigid substrates, which greatly increase process costs and make production unfeasible. While LLO improves precision, its limited throughput and reliance on costly ultrafast lasers render it unsuitable for large-area processing. An industrially promising alternative exploits the dangling-bond-free surfaces and weak interfacial bonds of 2D materials to enable gentle exfoliation with substrate reuse. Large-area synthesis and transfer of 2D materials are critical for scalability. Self-limiting growth modes that directly form large-area 2D materials on target substrates show particular promise for preserving surface integrity during this process. Notably, external forces and residual stresses during exfoliation may cause defects or cracks in epitaxial layers. Chemical lateral etching of sacrificial layers provides a damage-free substrate separation method. This low-cost approach is inherently suitable for large-area processing.

Furthermore, low-dimensional structures play a crucial role in enhancing device flexibility and optoelectronic performance through reduced Young’s modulus and increased surface-to-volume ratios. The direct growth of 1D micro/nanostructures on 2D layers represents an efficient pathway for fabricating high-performance flexible mixed-dimensional heterojunctions, avoiding transfer processes and scaling issues seen with other thin-film materials. Micro/nanodevices fabricated via top-down or bottom-up strategies can both be readily transferred to arbitrary flexible substrates using transfer printing. Micro/nanostructures prepared by the bottom-up strategy are especially advantageous as they inherently release strain during growth, eliminating residual stress that could compromise device performance during exfoliation. Combined with 2D material interfaces, this pick-and-place methodology with reusable soft stamps offers high manufacturability potential.

## Flexible optoelectronic applications

Advances in the growth and fabrication technology have significantly enhanced the performance and versatility of III-nitride flexible optoelectronics, sparking renewed interest in the field. As highlighted in Table 3, research into flexible optoelectronics has rapidly expanded, with applications spanning flexible displays, bioelectronics, flexible photodetectors, and mechanical sensing, etc.

**Table 3:** Flexible optoelectronic applications based on III-nitride semiconductors

Active materials	Flexible substrates	Device types	Flexible device implementation techniques	Applications	Features	Year
GaN thin films	Metal, glass, plastic	LED	Graphene-based transfer	Flexible lighting	First transferable LEDs	2010^16^
InGaN/GaN	Copper-plated plastic	LED	VdWE and transfer based on graphene	Flexible lighting	Operate reliably in a flexible form	2011^55^
InGaN	Plastic	LED	Anisotropic etching	Flexible lighting “tapes”	Minimize adverse thermal effects, easy to package and integrate	2011^64^
InGaN	Copper-PET	LED	Conducting adhesives	Backlights for flexible displays	Excellent stability during 100 forward and backward flexing cycles	2013^77^
GaN	Carbon-tape/graphene	LED	LLO	Flexible lighting	Graphene as a transparent-conductive layer	2014^65^
InGaN/GaN NWs	PET or metal foil	LED	Nanowire lift-off	Flexible lighting	Two-color emitting in the green and blue spectral ranges	2015^78^
InGaN	Ausn	LED	LLO and metal bonding	High brightness microdisplays	Enhanced thermal saturation current density and optical output	2016^66^
GaN	Copper foils	LED	SiO_2_ sacrificial layer etching	Flexible lighting	Reliable luminescence under extreme bending	2016^67^
InGaN/GaN	PI	LED	Hollow bases	Flexible or rollable displays	Ultra-small and ultra-high resolution pixels	2019^68^
GaN	Tungsten	LED	Micrographic fossil graphene	Flexible lighting	Streamlined LED microarray fabrication	2019^69^
InGaN/GaN	PET	LED	Transfer bonding technology	Flexible displays	Brightness levels of up to 5000 nits, color uniformity, and thermal stability	2020^70^
GaN	Copper	LED	CVD graphene	Flexible multicolor LED array	Stable EL and superior mechanical resilience under intense bending	2020^71^
GaN microrod	Copper	LED	RE and transfer based on graphene	Flexible displays	Deformable in various shapes without serious performance degradation	2020^21^
GaN	PET	LED	VdWE and Thermal-release tape	Flexible displays	High-brightness and high-stability	2021^72^
InGaN	Copper	LED	Self-lift-off and transfer	Flexible displays	100% crack-free transfer yield for the 400 tested LEDs	2021^73^
GaN	Copper	LED	Hybrid etching and fs-LLO	Flexible displays	High nanowire density, transfer yield, and reproducibility	2021^74^
GaN	mica	LED	Multiple exfoliated mica	Flexible displays	Reusable and reproducible III-nitride/mica structures	2024^61^
GaN	Copper	LED	VdWE and mechanical spalling	Flexible displays	66% luminescence enhancement with good reliability	2024^75^
GaN	Glass	LED	VdWE and transfer printing	Flexible displays	Peak intensities remain consistent before and after the transfer	2024^46^
GaN	Hydrogels	LED	Transfer printing	Bio-integrated sensors	Heat dissipation models with devices on hydrogels and other soft substrate	2012^80^
GaN	Parylene C	μ-LED	Exfoliate substrate and transfer print	Optogenetics	Wireless and programmable control over cellular activity	2013^82^
GaN	Copper foil	μ-LED	LLO, silver nanowire networks	Bilateral visual communication	High thermal stability, long lifetime, wirelessly powered, and implantable	2018^6^
GaN	Parylene C	μ-LED	Monolithically integrated with polymer	Optical neural probes	Wall-plug efficiency of 6.5% and an optical power output of 14.3 μW	2020^81^
GaN	Thin Sie	μ-LED	Suturing the Sie onto dura mater	Chronic optogenetic manipulation	High-throughput perturbation of neural activity with minimal invasiveness	2021^83^
GaN	PET	Photodetector	LLO and bonding	Flexible UV photoswitch	Self-powered, high on/off ratio and excellent sensitivity	2016^84^
GaN NWs	PI	NWs	Wet etching and transfer process	Flexible photodetectors	Photoresponsivity enhanced by the piezo-phototronic effect from GaN	2016^89^
AlGaN	Copper/PI	Photodetector	Substrate thinning and Bonding process	Wearable photodetector products	Photodetector integrated with a wireless module	2018^86^
GaN	Glass rods	Photodetector	VdWE and transfer based on graphene	Flexible UV photodetectors	Robust optoelectronic properties in UV on/off cycling tests	2019^85^
AlN	PEN	SAW	Multistep sputtering deposition	Flexible photodetectors	Photoresponse in the IR-Vis-UV range of a AlN piezoelectric SAW	2021^88^
AlGaN	PET/UV resin	Photodetector	VdWE and transfer based on high temperature nitridated graphene	foldable/wearable lighting sterilization and sensor	High device yield of 90% and excellent mechanical stability without any microscale structuration	2023^87^
InGaN/GaN	PMMA	Strain sensor	Nanopillar coated with PMMA	Dynamic pressure display	PL be modulated dramatically and linearly using the piezo-phototronic	2015^92^
InGaN/GaN	Multilayer graphene	Strain sensor	ZnO rods sacrificial template	Dynamic pressure distribution	Pressure-sensitive modulation of the emitting of nanopillars	2020^93^
AlN	PEN	SAW	Multistep sputtering deposition	Strain sensor	Passive wireless sensing deformation in critical and inaccessible points	2020^90^
InGaN/GaN	PDMS	Emitter/detector	Flexible bump layer	Three-axis tactile sensor	Small footprint, a high resolution of 2 mN, and high stability	2023^94^
GaN	Titanium	Thin-film sensor	Chemical etching Ni sacrificial layer	Wearable strain sensors	Enhanced built-in piezoelectric polarized field at the interface	2024^91^

### Flexible displays

Flexible displays surpass traditional counterparts by offering exceptional flexibility, softness, and extensibility, enabling next-generation applications in mobile devices, wearables, lighting, and instrumentation, such as foldable smartphones and in-car heads-up displays. As their functionality expands, they are expected to see widespread adoption in defense and commercial smart terminals. To meet this demand, flexible inorganic LEDs have emerged as key technologies. Compared to LCDs and OLEDs, III-nitride-based flexible displays offer higher brightness, improved dynamic range, and superior contrast.

**Fig. 9:**
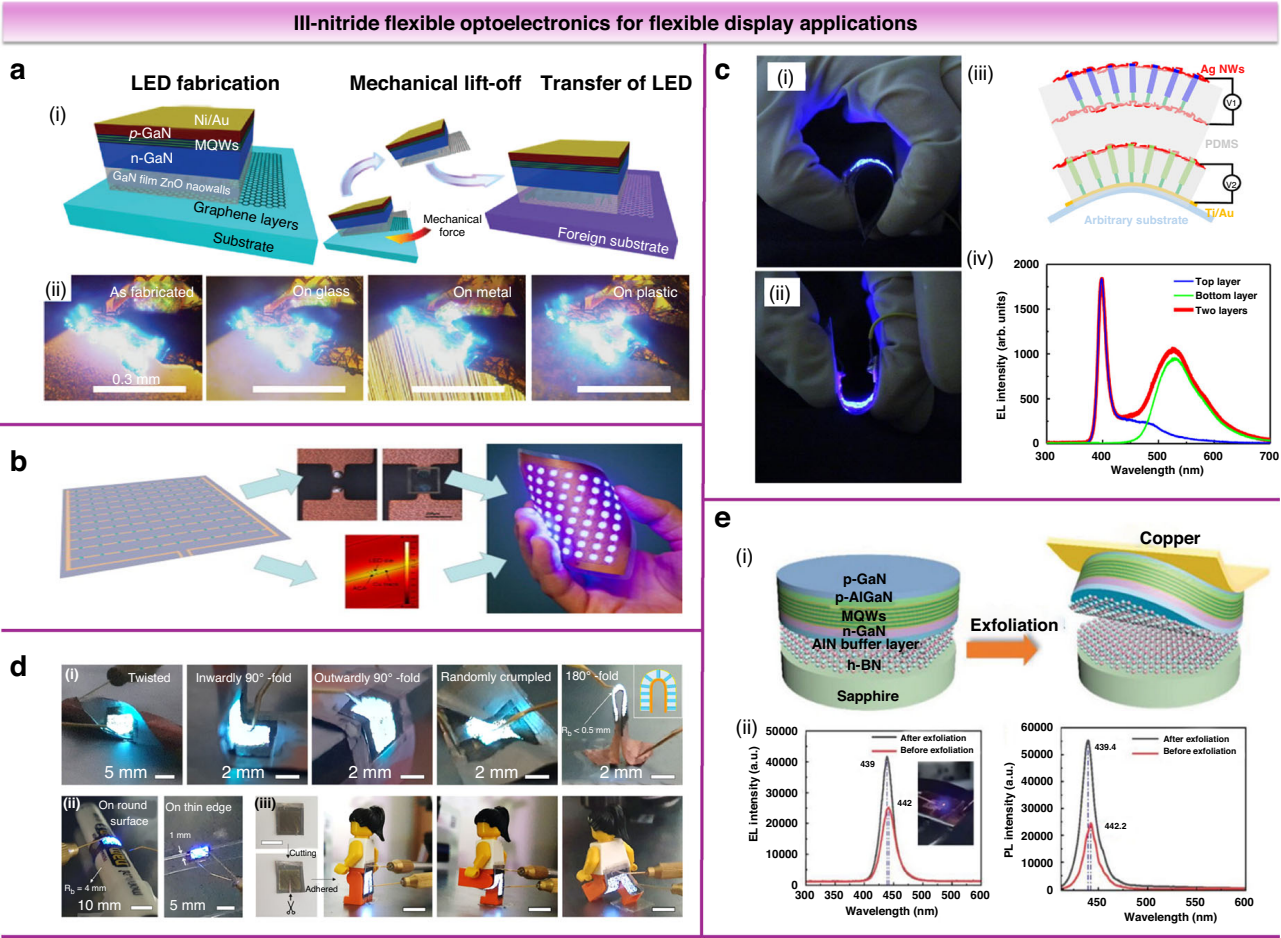
III-nitride flexible optoelectronics for flexible display applications. **a** Schematic illustration of fabrication and transfer processes for thin-film LEDs (ⅰ). Optical microscope (OMs) of light emissions from the as-fabricated LED on the original substrate and transferred LEDs on the foreign metal, glass, and plastic substrates (ⅱ). Reproduced with permission from ref. ^16^. Copyright 2010, The American Association for the Advancement of Science. **b** Process diagram and technology demonstrator of an 8×8 array of 8 mm spaced blue LED chips prepared on copper-PET foil. With permission from ref. ^77^. Copyright 2013, Elsevier. **c** Mechanical properties of the flexible LED (ⅰ, ⅱ), schematic of a two-color nanowire flexible LED (ⅲ), EL spectra of the emission from the top LED (blue curve), from the bottom LED (green curve), and a simultaneous emission from both LEDs (red curve) (ⅳ). Reproduced with permission from ref. ^78^. Copyright 2015, The Authors, published by American Chemical Society. **d** Photographs of flexible LED deformed in various shapes (ⅰ), and mounted on various surfaces (ⅱ), photographs of LED-adhered LEGO minifigure with different leg postures (ⅲ). Reproduced with permission from ref. ^21^. Copyright 2020, The Authors, published by American Association for the Advancement of Science. **e** Schematic illustration of the fabrication process for the flexible LED (ⅰ). EL spectra and PL spectra of the LED grown h-BN/sapphire before and after exfoliation (ⅱ). Reproduced with permission from ref. ^75^. Copyright 2023, Wiley-VCH GmbH

Research on III-nitride flexible LEDs began in 1999 when Wong et al. demonstrated free-standing InGaN LED membranes via LLO, showing stable device performance after removing rigid substrates^43^. Yi et al. introduced a graphene-layer transfer method for thin-film LEDs, enabling transfer to flexible substrates^16^. OMs' results (Fig. 9a) confirmed that LEDs transferred to metal, glass, and plastic substrates maintained strong electroluminescent (EL) emission. Since then, substantial progress has been made in III-nitride flexible LEDs using various substrates and transfer techniques (Table 3)^64–75^. To enhance flexible LED performance, the design has evolved into a micro-array format, enabling miniaturization, high resolution, high contrast, and mechanical flexibility^76^. III-nitride micro-arrays have thus emerged as promising flexible light sources. A demonstrator consisting of 8 × 8 flexible LED arrays was created by bonding LED dies onto polyethylene terephthalate (PET) substrates with copper circuitry using conductive adhesives (Fig. 9b)^77^. Dai et al. fabricated large-area flexible blue LEDs using InGaN/GaN NWs grown by metal-organic vapor phase epitaxy^78^. These LEDs maintained stable EL under repeated bending to a 3 mm radius and operated for over a month in ambient conditions without encapsulation. The study also demonstrated fully transparent flexible LEDs and achieved two-color emission by integrating InGaN/GaN NW layers with different indium contents, covering green and blue spectra (Fig. 9c). Jeong et al. achieved heterogeneous epitaxy of GaN microrod PN junction arrays on graphene, followed by transfer onto copper plates^21^. The spatial separation of microrod LEDs enabled deformable panels in various shapes while maintaining performance under significant deformation. Figure 9d shows flexible LEDs conforming to surfaces of different curvatures and stabilizing in various minifigure leg positions. Enhancing post-transfer device performance remains crucial. Wang et al. grew high-quality metastable GaN thin films on an AlN/h-BN composite buffer layer^75^. The study proposed a strain-relaxation model, where a high-temperature AlN buffer induces tensile strain during rapid coalescence, compensating for heteromismatch-induced compression. The flexible LEDs exhibited a ~ 66% luminescence enhancement post-transfer with high reliability (Fig. 9e), providing a solid foundation for flexible displays.

**Fig. 10:**
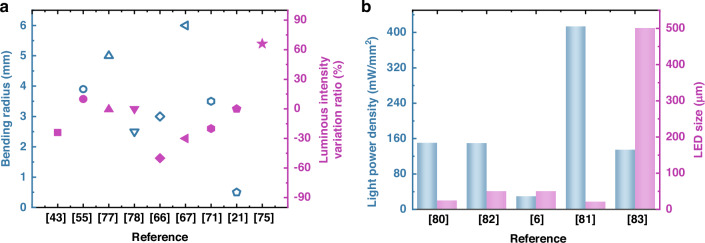
Performance comparisons of III-nitride LEDs for flexible applications. **a** A summary of the reported bending radius of III-nitride flexible LEDs and changes in luminous intensity after transfer or bending^21,43,55,66,67,71,75,77,78^. **b** Maximum light output power density and sizes for implantable III-nitride micro-LEDs^6,80–83^

As illustrated in Fig. 10a, state-of-the-art III-nitride flexible LEDs demonstrate fine flexibility with a minimum bending radius of 0.5 mm^21^. These devices do not deteriorate significantly after bending, while some exhibit significantly enhanced luminescence intensity post-transfer due to strain release effects^75^. Although III-nitride micro-array flexible displays have advanced, they still lag behind organic flexible displays in performance. Achieving full-color micro-LED displays remains a challenge due to limitations in single-growth and sequential transfer methods. Color conversion using organic/inorganic phosphors or quantum dots offers a promising alternative, with quantum dots enabling smaller pixel sizes as pitch decreases^79^.

### Implantable bioelectronics

**Fig. 11:**
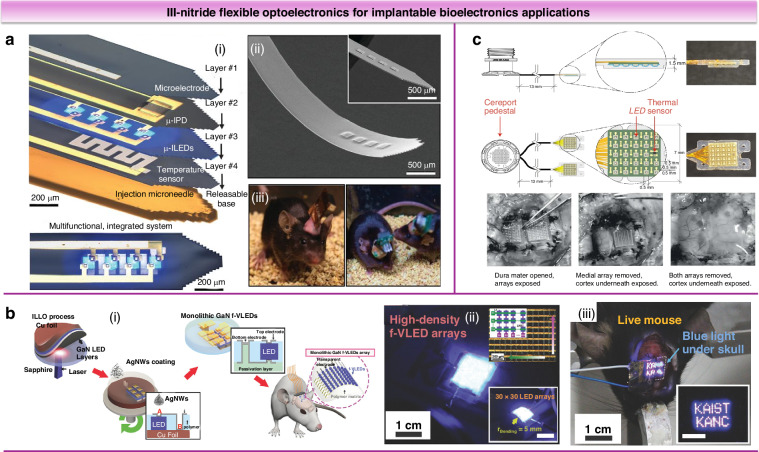
III-nitride flexible optoelectronics for implantable bioelectronics applications. **a** Schematic of the multifunctional, implantable optoelectronic system (ⅰ), SEM of an injectable array of μ-ILEDs (ⅱ), a custom flexible polyimide film–based scavenger, or a rigid printed circuit board–based scavenger mounted on freely moving animals (ⅲ). Reproduced with permission from ref. ^82^. Copyright 2013, American Association for the Advancement of Science. **b** Schematic illustration of the fabrication procedure and biomedical application of the monolithic transparent GaN f-VLED (ⅰ), a picture of high-density monolithic f-VLED arrays (ⅱ), and a picture of a head-fixed, living mouse with the f-VLED array (ⅲ). Reproduced with permission from ref. ^6^. Copyright 2018, Wiley-VCH GmbH. **c** Schematic of the Opto-Array design and photographs of the brain surface after explantation of the Opto-Array after 155 days implanted in a monkey. Reproduced with permission from ref. ^83^. Copyright 2021, Springer Nature

III-nitride materials’ excellent biocompatibility, stability, and favorable optoelectronic and surface properties make them ideal for biomedical applications^80^. In optogenetics, III-nitride LEDs precisely activate opsins, light-sensitive proteins introduced into cells for optical control of cellular activity. As shown in Fig. 10b, the micro-LEDs integrated into the optogenetic system have micrometer-scale dimensions (minimum 22 × 22 μm)^81^ and sufficiently high light power density to enable precise studies of neural circuits without tissue damage. A pioneering study by Kim et al. developed injectable, cellular-scale GaN μ-LEDs for optogenetics, enabling precise positioning within biological tissues^82^. These ultrathin, biocompatible devices operated minimally invasively and have been demonstrated in freely moving animals, allowing wireless, programmable control of cellular activity (Fig. 11a). Fabricated from biocompatible Parylene C, they conform to tissues, reducing damage and immune response, offering a promising platform for untethered optogenetic modulation. Lee et al. developed monolithic flexible vertical GaN LEDs (f-VLEDs) on a silver nanowire network for wireless brain optical stimulation (Fig. 11b)^6^. These transparent f-VLEDs achieved a projected 12-year lifespan and exceptional thermal and mechanical stability, enduring over 100,000 bending cycles. Successfully integrated into a wireless skin-applied system, high-density f-VLED arrays implanted in the mouse cortex operated effectively without histological damage, demonstrating their biocompatibility for in vivo optogenetics. Reddy et al. developed flexible neural probes integrating GaN micro-LED arrays on Parylene C substrates for optogenetic stimulation^81^. Multiphysics modeling verified operational safety, maintaining tissue temperature rise <1 °C during 5 ms pulsed operation. Rajalingham et al. developed the Opto-Array, an implantable LED array for chronic optogenetic manipulation in primates(Fig. 11c)^83^. It enabled high-throughput neural perturbation with minimal invasiveness and reliably induced visual deficits in macaques through optogenetic silencing in the primary visual cortex. The device maintained stable light output for 155 days, with thermal testing confirming neural modulation rather than tissue heating. This advancement expands optogenetics to larger brains, offering a powerful tool for studying sensory, motor, and cognitive functions in primates.

The integration of flexible GaN LED arrays into optogenetic devices represents a promising avenue for advancing neural stimulation and sensing, with the potential to unlock novel applications in neuroscience and biomedical research. The development of these innovative devices highlights the critical need for continued advancements in material processing and optoelectronic integration to fully harness the capabilities of GaN LEDs in the field of optogenetics.

### Wearable photodetectors

**Fig. 12:**
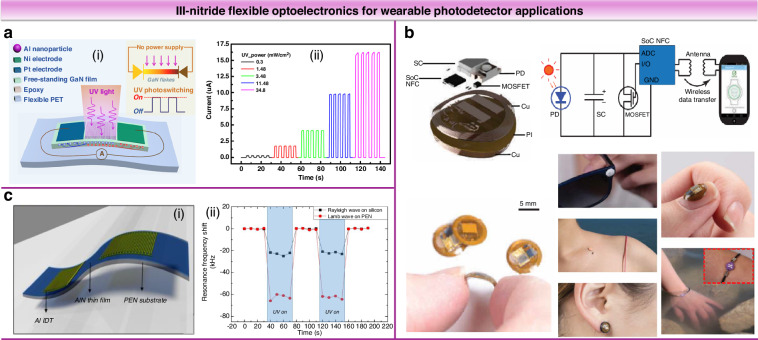
III-nitride flexible optoelectronics for wearable photodetector applications. **a** Schematic diagram of a self-powered GaN UV photoswitch (ⅰ), and its self-powered on/off switching response as a function of the UV light power at 0 V bias (ⅱ). Reproduced with permission from ref. ^84^. Copyright 2016, American Chemical Society. **b** Schematic, circuit diagram, and application in body parts of millimeter-scale, battery-free, wireless sensors of UVA radiation. Reproduced with permission from ref. ^86^. Copyright 2018, The American Association for the Advancement of Science. **c** 3D schematic of the AlN-based SAW device on flexible PEN substrate (ⅰ). Real-time resonance shift of Lamb and Rayleigh modes in AlN SAW UV detector under 365 nm LED source (ⅱ). Reproduced with permission from ref. ^88^. Copyright 2021, IEEE

III-Nitride-based flexible photodetectors are crucial for UV sensing, offering protection against UV-induced hazards such as sunburn, inflammation, DNA damage, and increased skin cancer risk. The development of these photodetectors supports real-time, wearable UV monitoring, providing continuous protection and timely intervention against harmful exposure^32^. Peng et al. developed a flexible GaN UV photoswitch with an asymmetric metal-semiconductor-metal structure to enhance carrier separation and transport (Fig. 12a)^84^. This device achieved a high on/off ratio of 4.67 × 10^5^ and sensitivity of 1.78 × 10^12 ^cm∙Hz^0.5^ W^1-^. The study explored the piezo-phototronic effect on UV detection, achieving a self-powered operation with a 154% increase in switching ratio. Liu et al. fabricated flexible UV photodetectors by mechanically exfoliating and transferring a single-crystal GaN film on graphene/SiC substrates^85^. These detectors maintained stable performance across 2.5 mm bending radius and demonstrated good sensitivity and UV on/off ratio of 167%. Heo et al. developed a self-powered, flexible AlGaN UV photodetector with an integrated wireless module, whose output voltage is linearly related to the exposure dose of UVA and UVB. This compact device can be used on the skin surface as well as in various scenarios such as sunglasses, nails, and earrings (Fig. 12b)^86^. It is now commercially available worldwide, highlighting the potential of smart wearable photodetectors. Chen et al. prepared flexible photodetectors utilizing monolayer graphene as a photocarrier transport channel, showing up to 90% device yield and excellent mechanical stability^87^. After 250 bending cycles, the flexible photodetector maintained about 60% of its UV responsivity of 0.239 A ∙ W^-1^. Specifically, Lamanna et al. developed a flexible AlN-based piezoelectric SAW photodetector (Fig. 12c) utilizing photovoltaic mechanisms, which exhibited UV photoresponse through frequency downshift of the Rayleigh and Lamb resonances modes, advancing flexible optoelectronic device engineering^88^.

In addition to UV spectrum applications, bandgap-tunable III-nitride materials have also been used in flexible photodetectors for other spectral bands. Liu et al. fabricated FETs with a single layer of MoS_2_, where GaN NWs acted as localized gates. Strain applied to the GaN NWs enhanced the optical responsivity of 443.3 A ∙ W^-1^ at 550 nm via the piezo-phototronic effect^89^.

### Flexible mechanical sensing

**Fig. 13:**
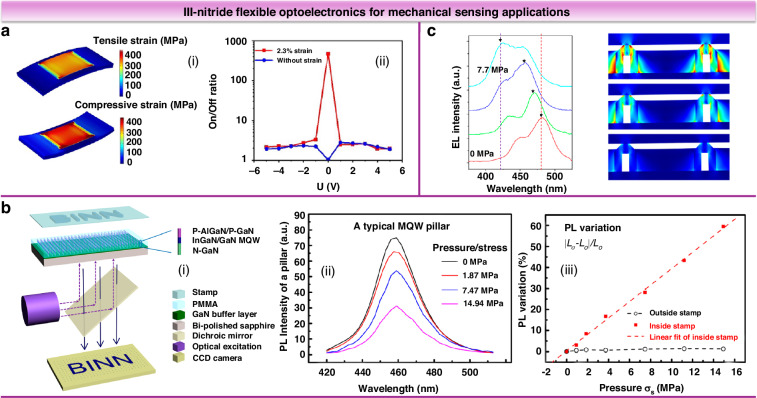
III-nitride flexible optoelectronics for mechanical sensing applications. **a** Stress distribution of the sensor under bending (ⅰ). The ratio of light/dark current under tensile strain and without strain (ⅱ). Reproduced with permission from ref. ^91^. Copyright 2024, IOP Publishing Ltd. **b** Schematic diagram of piezo-phototronic effect tuned PL imaging (ⅰ). PL spectra (ⅱ) and PL intensity (ⅲ) under various compressive stresses. Reproduced with permission from ref. ^92^. Copyright 2015, American Chemical Society. **c** EL spectra and FEA simulation of the strain distribution under external stress increased from 0 to 7.7 MPa. Reproduced with permission from ref. ^93^. Copyright 2020, American Chemical Society

Flexible mechanical sensors are a prominent research area with significant market value, finding applications in health monitoring, wearable devices, and intelligent robotics. Leveraging the principles of piezotronic and piezo-phototronic effects, III-nitride devices have facilitated the development of flexible mechanical sensors^90^. Chen et al. developed a flexible mechanical sensor using a piezoelectric polarized interface modulation in GaN/Ti Schottky structures^91^. The current was reduced by 53.9% at 2.3% tensile strain and enhanced by 67.8% at 2.3% compressive strain. It was also observed that the light/dark current ratio increased under tensile strain up to 471 (Fig. 13a). Peng et al. reported a dynamic pressure sensor array utilizing the piezo-phototronic effect, comprised of InGaN/GaN multiple quantum well nanopillars^92^. Small strain (0–1.15%) induced piezoelectric charges modulated PL intensity linearly and at ultra-high speeds (Fig. 13b). The resultant all-optical pressure sensing arrays featured high pixel density (6350 dpi) and exhibited minimal standard deviation, facilitating large-area dynamic pressure sensing. Yang et al. leveraged crystal strain-induced wavelength shifts for pressure optical monitoring^93^. Using a hollow-core design, 3D InGaN/GaN microcrystals exhibited a notable 50 nm wavelength shift under 8 MPa stress (Fig. 13c). Integrating III-nitride strain sensors with other functional units could further enhance performance. Yin et al. designed and fabricated a flexible GaN optical three-axis tactile microsensor by monolithically integrating a light emitter with four light-detecting units^94^. The sensor, enhanced by a PDMS bump layer, was capable of responding effectively to both normal and shear forces, exhibiting a high resolution of 2 mN at 50–200 mN range.

III-nitride flexible mechanical sensors offer high sensitivity and resolution, but further research is needed to improve their dynamic response, including hysteresis, response time, signal drift, and strain rate dependence. Balancing sensitivity and sensing range also requires investigation. In practical applications, mechanical forces often combine pressure, tension, shear, and torsion, as seen in robotics, gesture recognition, and prosthetics. Thus, multimodal sensing is a key goal, but decoupling these stimuli remains a challenge.

In addition to the aforementioned applications, many III-nitride devices originally fabricated on rigid substrates have been adapted for flexible optoelectronics, enhancing performance and expanding their application range. These include flexible photocatalysts^58,95^, piezo-phototronic effect-enhanced solar cells^96^, and visible light communication devices^97,98^.

## Conclusion and perspective

III-nitride semiconductors, with their exceptional optoelectronic, piezotronic, and piezo-phototronic properties, as well as biocompatibility and stability, have emerged as advanced materials capable of addressing the challenges faced by traditional organic and Si-based flexible optoelectronics. Developments in material growth and preparation techniques were driving the evolution of III-nitride devices towards flexible optoelectronics. Advances in vdWE and RE technologies offer more substrate options for heterogeneous epitaxy. The realization of film exfoliating and transfer has made the III-nitride flexible optoelectronics possible. Functional micro/nanostructures provide the mechanical flexibility necessary for flexible devices. Material properties combined with evolving fabrication methods drive the widespread adoption of III-nitride flexible optoelectronics. High-brightness and contrast flexible LEDs, with wide spectral and dynamic ranges, are ideal for next-gen flexible displays and light communications. Due to the biocompatibility and stability, III-nitride bioelectronics are promising for implantable optogenetic devices. Bandgap-tunable III-nitride materials have been used in flexible photodetectors for various spectral. The strong piezoelectric effect enables flexible mechanical sensors to achieve high sensitivity and resolution. While some devices, such as AlGaN-based UV flexible photodetectors, have been commercialized in wearable forms, significant challenges remain for III-nitride flexible optoelectronics before they can compete with the market dominance of organic and Si-based products. Section 3.4 discusses technical challenges in device fabrication. Here, we summarize key challenges in tolerating mechanical damage, enhancing device performance, and integrating functional systems, along with proposed solutions.

1) The primary challenge for III-nitride flexible devices lies in maintaining structural integrity and performance under unpredictable mechanical interactions with the environment. Although III-nitride semiconductors achieve excellent flexibility through thickness reduction and micro/nano-structuring, their transfer onto flexible substrates creates an interfacial mechanical mismatch. Under extreme stresses, including large strains, high-impact loads, prolonged cycling, or sustained friction, stress concentrations at heterogeneous material interfaces can induce cracking/delamination. This critically degrades mechanical/thermal signal transfer at interfaces, compromising signal stability and heat dissipation.

2) Most III-nitride flexible optoelectronic devices are in early research stages. Although performance has been demonstrated on flexible substrates, these devices typically offer a single sensing mode and lack comprehensive studies on dynamic response, selectivity, and sensitivity. Flexible applications require extensive stability testing due to complex environmental stresses. Additionally, research on the theoretical mechanisms behind device stability is limited.

3) Simultaneously responding to mechanical, electrical, optical, and magnetic stimuli in a single material is nearly impossible. Device versatility often requires coupling multiple physical properties, emphasizing the need for integrating diverse components in future flexible optoelectronics. While monolithic integration of III-nitride devices is still in its early stages, overcoming substrate limitations for heterogeneous multi-material integration remains a key challenge.

To address the outlined challenges, we have proposed several strategies and recommendations:

1) To enhance mechanical reliability, four strategies are proposed: (ⅰ) Stress-relief structural engineering: device flexibility, governed not only by materials but also geometric designs. Structures like wavy, origami-inspired, microcracked, or honeycomb can maximize elastic stretchability by converting macroscopic tension into localized bending/torsion. (ⅱ) Graded stiffness transitions: buffer layers with intermediate Young’s modulus between rigid active layers and soft substrates can progressively dissipate strain energy. (ⅲ) Microstructured interface engineering: reducing contact area via elastomer contact points or embedded rigid islands suppresses crack propagation, extending failure strain and fatigue life. (ⅳ) Advanced encapsulation: embedding fragile active layers within hydrogel matrices or viscoelastic polymers enhances overall stability without impeding optoelectronic functionality.

2) Future device research should explore multi-modal sensing mechanisms to enhance device functionality through the integration of diverse sensing units or modes. Enhanced efforts are needed in multi-dimensional stability testing, addressing environmental factors including time, stress, temperature, and humidity. Additionally, building on piezo-phototronic theory, further research should focus on enhancing device performance through strain. Furthermore, developing comprehensive physical models for flexible systems, including force-electric coupling, thermal management, and stress-induced failure, is essential. Integrated device models will support III-nitride flexible optoelectronics circuit design.

3) Integrated systems can incorporate various sensors through matrix networks or stacked architectures, allowing for the full utilization of III-nitride material advantages via heterogeneous integration. Combining III-nitride materials with inorganic substrates like Si, as well as integrating inorganic and organic devices through flexible substrates, is highly significant. Additionally, the integration of signal processing circuits, such as differential amplifiers and adaptive filters, with the devices is also necessary. Improved sensor architectures, along with compensation algorithms, feedback, and drive circuits, can mitigate issues like noise and temperature drift. Ultimately, the goal is to create flexible optoelectronic systems that integrate sensing, signal processing, communication, and control functions, driving the future development of III-nitride flexible optoelectronics.
